# Dietary habits as associated factors with irritable bowel syndrome among medical students: evidence from a cross-sectional study

**DOI:** 10.1186/s12876-024-03320-w

**Published:** 2024-08-17

**Authors:** Mueataz A. Mahyoub, Osama Abbas, Mohamed Elhoumed, Saif Ghabisha, Moath Refat, Mustafa Abbas, Sarah Al-Qurmoti, Yarui Li, Mudan Ren, Shuixiang He

**Affiliations:** 1 Department of Gastroenterology, The First Affiliated Hospital of Xi’an Jiaotong University, Xi’an, 710061 China; 2 Clinical Medical Research Center for Digestive Diseases (Oncology) of Shaanxi Province, Xi’an, 710061 China; 3 Department of Gastroenterology, Faculty of Medicine, Thamar University, Thamar, Yemen; 4 Department of Human Medicine, Faculty of Medicine, Jibla University for Medical and Health Sciences, Jibla, Yemen; 5 Department of Epidemiology and Biostatistics, School of Public Health, Xi’an Jiaotong University Health Science Center, Xi’an, Shaanxi China; 6 Department of Epidemiology, National Institute of Public Health Research (INRSP), Nouakchott BP. 695, Nouakchott, Mauritania; 7 Department of General Surgery, Faculty of Medicine, Ibb University, Ibb, Yemen; 8 Department of Biochemistry and Molecular Biology, The Key Laboratory of Environment and Genes Related to Disease of Ministry of Education, Health Science Center, Xi’an Jiaotong University, Xi’an, China; 9 Department of Internal Medicine, Faculty of Medicine, Thamar University, Thamar, Yemen

**Keywords:** Dietary habits, Irritable bowel syndrome, Rome IV criteria, Yemeni medical students

## Abstract

**Background:**

Research on Irritable Bowel Syndrome (IBS) among medical students has increased globally, highlighting a high prevalence in this demographic. However, there is a lack of data specifically regarding the prevalence of IBS among medical students in Yemen. This study aimed to investigate the prevalence and associated factors of IBS among Yemeni medical students.

**Methods:**

We conducted a cross-sectional study involving medical students who completed a validated self-administered questionnaire incorporating socio-demographic information, dietary habits, smoking status, sleep patterns, and the Rome IV criteria for IBS. We used bivariate and multivariate logistic regression models to identify IBS’s associated factors, estimated as odds ratios (ORs) with 95% confidence intervals (CIs) and average marginal effect (AME) on the predicted probability of IBS.

**Results:**

The study included 351 medical students with a mean age of 22.53 ± 2.70 years; 39.60% (139) were females. The prevalence of IBS was 26.21% (92 students), with 67.39% (62) of them classified as IBS-M (mixed). In multivariable analysis, the consumption of carbonated soft drinks remained significantly associated with IBS (OR: 3.35; 95% CI: 1.14–9.88; *P* = 0.028). In males, coffee consumption had a substantial effect on the predicted probability of IBS (AME: 11.41%; 95% CI: 0.32–22.60). In females, the consumption of carbonated soft drinks had a significant effect on the predicted probability of IBS (AME: 24.91%; 95% CI: 8.34–41.48).

**Conclusion:**

The consumption of carbonated soft drinks is significantly associated with IBS among medical students, with a particularly notable increase in the predicted probability of IBS in females. These findings highlight the necessity for gender-specific dietary recommendations in IBS management. Further research is essential to investigate IBS in the general population to gain a comprehensive understanding of its prevalence and associated factors.

**Supplementary Information:**

The online version contains supplementary material available at 10.1186/s12876-024-03320-w.

## Background

Irritable bowel syndrome (IBS) is recognized as a highly prevalent and economically impactful gastrointestinal disorder [1, 2]. While it doesn’t lead to increased mortality, its socioeconomic impact is profound, diminishing the quality of life for those affected, necessitating additional healthcare resources, and affecting productivity. IBS is characterized as a chronic gut-brain disorder presenting with altered bowel habits, abdominal discomfort, or pain, all in the absence of organic pathology [3].

Although significant research has been done, the pathophysiological mechanisms of IBS have yet to be fully understood [4]. It is widely accepted that the disorder arises from a dynamic interaction of various factors—dietary patterns, psychological stress, gut microbiota alterations, and genetic predispositions. Environmental influences and lifestyle choices also play pivotal roles in the manifestation and exacerbation of IBS symptoms [5].

The prevalence of IBS varies worldwide, influenced by geographic factors and the criteria used for diagnosis. In a global analysis of six studies employing the Rome IV criteria, encompassing 34 countries and 82,476 individuals, the prevalence of IBS was found to be 3.8% (95% CI: 3.1–4.5%; I²=96.6%) [6].

Among medical students, the prevalence of IBS ranged from 9.3 to 35.5% when diagnosed using the Rome III criteria [7]. Crucially, there is no definitive diagnostic test or biomarker for IBS, and its diagnosis relies solely on clinical assessment. The criteria for diagnosing IBS have evolved over time, from the Manning criteria in the 1970s to the current Rome IV criteria [8].

Medical students encounter distinctive challenges relative to other university students. They consistently experience high levels of stress, extended study hours, substantial workloads, sleep deprivation, elevated caffeine consumption, and difficulties in maintaining their social lives [9, 10]. In Yemen, the convergence of societal stressors with ongoing political instability and infrastructural deficits amplifies the relevance of studying IBS among medical students [11]. Yemeni medical students face a unique array of challenges, including the demanding task of mastering a complex curriculum in a non-native language and managing the financial burden of their education. These factors create a distinctive context that may significantly influence the prevalence and characteristics of IBS within this group, underscoring the importance of this study in understanding and addressing the condition in such a challenging environment. Previous studies reported that fewer males have IBS compared to females [6, 12]. Despite the global increase in research on IBS among medical students, the prevalence and associated factors specific to Yemeni medical students, as well as the sex-specific effects of these factors, such as dietary habits on the IBS, remain understudied. This research aimed to fill this gap by studying the prevalence of IBS and its associated factors among medical students at Ibb University, Yemen. Additionally, it aimed to investigate the factors associated with IBS, stratified by sex.

## Methods

### Study design and setting

This cross-sectional observational study was carried out from September 2022 to March 2023 among students enrolled at the Faculty of Medicine, Ibb University, Ibb governorate, Yemen. This time frame was selected to accommodate academic schedules and ensure maximum student participation.

### Participants

Our study encompassed medical students of Ibb University from the first to the sixth year. We included all full-time medical students at the university during the study period and provided informed consent. We excluded the female students who reported abdominal pain or discomfort related to the menstrual cycle, students who reported organic gastrointestinal disorders, a family history of cancer, inexplicable weight loss, anaemia, bloody stools, abnormal laboratory results, other alarming signs, and individuals with a history of gastrointestinal surgery.

### Sample size

The sample size was calculated using the subsequent equation: [n = Z^2^ × P × (1-P) / d^2^], with a 95% confidence interval, power of 80%, and precision of 0.04, assuming a prevalence of 15% [13].

Where:

Z = 1.96 (Z-score for a 95% confidence level)

*P* = 15% (estimated prevalence of IBS)

Q = 1 - P

N = PQ / (SE^2^) = PQ / (d/Z)^2^ = (Z^2^ × PQ) / d^2^

Substituting the values:

$${\rm{N}}\,{\rm{ = }}\,{\rm{((1}}{\rm{.96}}{{\rm{)}}^2}\, \times \,{\rm{0}}{\rm{.15}} \times (1 - 0.15))/{(0.04)^2}\, \approx \,306.34$$

To ensure reliability, we enrolled 351 medical students in the study.

### Data collection and definitions of IBS and other covariates

Data collection was executed through a structured and validated self-assessment questionnaire. For efficient administration and clarification of the questionnaire content, a student from each academic year was recruited and trained. Their role was to elucidate the study’s purpose and assist in data gathering. The questionnaire consisted of two parts. The first part collected socio-demographic and lifestyle information (supplementary file 2), including age, sex, academic year, body mass index (BMI), employment status (employed or not), and living arrangements (with family or independently).

Additionally, it explored sleep patterns (early or late), dietary habits, food preferences (including fast food, fatty food, and legumes), beverage consumption (carbonated soft drinks, tea, and coffee), and smoking status (current smoker or not). Students were categorized based on their year in the medical program: junior (first three years) and senior (fourth year onwards). We categorized students as coffee drinkers, tea drinkers, and carbonated soft drink drinkers based on their daily consumption of these beverages. Additionally, we categorized eating patterns as either quick, characterized by rapidly finishing a meal, or slow, involving the unhurried enjoyment of meals at a more relaxed pace.

The second part included the Arabic version of the Rome IV criteria questionnaire [3, 14], which diagnoses IBS based on the presence of recurrent abdominal pain/discomfort for at least one day per week during the past three months, persisting for at least six months, along with two or more of the following: (i) the pain’s relationship to defecation, (ii) a change in stool frequency, and (iii) a change in stool appearance. The Rome IV Criteria for IBS diagnosis have an excellent specificity of 97.1% and a moderate sensitivity of 62.7% [15]. Considering the self-assessment format of the study questionnaire, the second part concluded with binary ‘yes’ or ‘no’ questions designed to identify and exclude red flag symptoms as outlined in the exclusion criteria. The subtypes of IBS were categorized based on stool consistency as follows: IBS with constipation (IBS-C) was characterized by experiencing hard or lumpy stools in the majority of bowel movements. Conversely, IBS with diarrhoea (IBS-D) involves having loose or watery stools in the majority of bowel movements. Mixed IBS (IBS-M) was identified when individuals experienced a mix of hard or lumpy and loose or watery stools, occurring with similar frequencies over time. Un-subtyped or unknown IBS (IBS-U) was used to describe cases where stool consistency did not consistently align with the patterns of IBS-C, IBS-D, or IBS-M, indicating a lack of sufficient deviation in stool consistency to meet the criteria of these subtypes [16].

### Statistical analysis

Statistical analysis was conducted utilizing STATA version 17.0. General characteristics were presented using descriptive statistics, including total counts and percentages. Group comparisons were assessed using either Pearson’s χ² test or Fisher’s exact test, as appropriate. Continuous variables were evaluated using t-tests, wherein findings were displayed using mean values and standard deviations (SD). Binary logistic regression analysis was employed to identify independent associations with IBS in comparison to the control group without IBS [17]. The findings were reported as odds ratios (OR) with corresponding 95% confidence intervals (CI). We explored the average marginal effects (AME) in the predicted probability of developing IBS, adjusted for confounders included in the multivariable model. AME was calculated separately for males and females to elucidate potential sex-specific effects of significant predictors. All p-values reported in the study were calculated using a two-tailed test, and statistical significance was determined as *p* < 0.05.

## Results

### Characteristics of the study population

Out of 450 questionnaires distributed, 351 participants responded, resulting in a response rate of 78%. Among these participants, 60.40% (212) were males, and 39.60% (139) were females. The mean age of the respondents was 22.53 years (SD 2.70). Furthermore, 55.84% (196) of the participants were juniors, while 44.16% (155) were seniors (Table 1).

**Table 1:** General characteristics of the population

Variables	Overall	IBS (n = 92) 26.21%	Non-IBS (n = 259) 73.79%	*p*-value
Age year, mean (± SD)	22.53 (2.70)	22.73 (2.09)	22.46 (2.88)	0.4125⚫
Gender				0.034*
Male, n (%)	212 (60.40)	47 (22.17)	165 (77.83)	
Female, n (%)	139 (39.60)	45 (32.37)	94 (67.63)	
Academic grade, n (%)				0.041*
Junior	196 (55.84)	43 (21.94)	153 (78.06)	
Senior	155 (44.16)	49 (31.61)	106 (68.39)	
BMI, kg/m2, mean (± SD)	21.49227 (8.12)	21.08 (3.81)	21.64 (9.18)	0.5694⚫
Smoking, n (%)				0.862*
Yes	36 (10.26)	9 (25.00)	27 (75.00)	
No	315 (89.74)	83 (26.35)	232 (73.65)	
Living with family, n (%)				0.014*
Yes	210 (59.83)	65 (30.95)	145 (69.05)	
No	141 (40.17)	27 (19.15)	114 (80.85)	
Eating style, n (%)				0.104*
Eaten at home	192(54.70)	57 (29.69)	135 (70.31)	
Eaten out home	159 (45.30)	35 (22.01)	124 (77.99)	
Sleep hours, mean (± SD)	6.39 (1.40)	6.40 (1.24)	6.39(1.46)	0.9338⚫
Sleep pattern, n (%)				0.310*
Sleep early	94 (26.86)	21 (22.34)	73 (77.66)	
Sleep late	256 (73.14)	71 (27.73)	185 (72.27)	
Working, n (%)				0.393*
Yes	48 (13.67)	15 (31.25)	33 (68.75)	
No	303(86.33)	77 (25.41)	226 (74.59)	
Fast food, n (%)				0.532*
Yes	315) 89.74)	81 (25.71)	234 (74.29)	
No	36 (10.26)	11 (30.56)	25 (69.44)	
Fatty food, n (%)				0.084*
Yes	206 (58.69)	61 (29.61)	145 (70.39)	
No	145 (41.31)	31 (21.38)	114 (78.62)	
Eat legumes, n (%)				0.400*
Yes	325 (92.59)	87 (26.77)	238 (73.23)	
No	26 (7.41)	5 (19.23)	21 (80.77)	
Carbonated soft drinks, n (%)				**0.019#**
Yes	312 (88.89)	88 (28.21)	224 (71.79)	
No	39 (11.11)	4 (10.26)	35 (89.74)	
Drink tea, n (%)				0.177#
Yes	332 (94.59)	90 (27.11)	242 (72.89)	
No	19 (5.41)	2 (10.53)	17 (89.47)	
Drink coffee, n (%)				
Yes	288 (82.05)	83 (28.82)	205 (71.18)	0.017*
No	63 (17.95)	9 (14.29)	54 (85.71)	
Eating pattern				
quick	139 (39.60)	39(28.06)	100 (71.94)	**0.524**
slow	212 (60.40)	53(25.00)	159 (75.00)	

⚫Two-sample t test

*Person chi-squared test

# Fisher’s exact test

The bold values signify statistically significant results, typically with a p-value less than 0.05

### Prevalence of IBS and characteristics of students with and without IBS

The prevalence of IBS in the study population was 26.21% (92), and the predominant type was mixed bowel habit (IBS-M), 67.39% (62) of IBS patients. IBS with diarrhoea (IBS-D) accounted for 23.91% [22], and IBS with constipation (IBS-C) made up 8.70% [8] of the IBS patients.

The female students exhibited a significantly higher IBS prevalence (32.37%) compared to their male counterparts (22.17%). The Senior students displayed a notably significantly increased prevalence of IBS (31.61%) compared to junior students (21.94%). The students living in their family homes showed a significantly higher prevalence of IBS (30.95%) compared to students living independently (19.15%). The students who consumed carbonated soft drinks had a significantly higher IBS prevalence (28.21%) compared to non-consumers (10.26%). Similarly, students who consumed coffee exhibited a significantly higher IBS prevalence (28.82%) in contrast to non-consumers (14.29%).

### Association between covariates and IBS

In the univariate analysis, major variables were investigated for their potential associations with the prevalence of IBS. Age showed a marginally non-significant association with IBS, where participants aged 22 years or older had 57% higher odds of having IBS compared to their younger counterparts (odds ratio [OR] = 1.57, 95% CI: 0.98–2.54). The female gender exhibited 68% higher odds of IBS compared to males (OR = 1.68, 95% CI: 1.04–2.72, *p* = 0.034). The senior students showed significantly higher odds of having IBS compared to juniors (OR = 1.64, 95% CI: 1.02–2.65, *p* = 0.042). The living arrangement showed a protective association, where individuals living without family had lower odds of IBS (OR = 0.53, 95% CI: 0.317–0.88, *p* = 0.014). Regarding dietary habits, coffee consumption was significantly associated with higher odds of IBS (OR = 2.43, 95% CI: 1.15–5.14, *p* = 0.020) when compared to non-consumers. Similarly, consumption of carbonated soft drinks was strongly associated with higher odds of IBS (OR = 2.43; 95% CI: 1.15–5.14, *p* = 0.023) in contrast to non-consumers (Table 2). Other factors were not found to be significantly associated with IBS.

**Table 2:** Univariate and multivariate analysis of the association between IBS and participant’s characteristics

Variables	Categories	Bivariate (Unadjusted)			Multivariate (Adjusted^1^)		
		OR	95% CI	*P* -value	OR	95% CI	*P* -value
Age (year)	< 22	1	[Reference]	0.063	1	[Reference]	0.290
	≥ 22	1.57	0.98–2.54		1.40	0.75–2.62	
Sex	Male	1	[Reference]	**0.034**	1	[Reference]	0.259
	Female	1.68	1.04–2.72		1.37	0.79–2.37	
Academic grade	Junior	1	[Reference]		1	[Reference]	
	Senior	1.64	1.02–2.65	**0.042**	1.18	0.63–2.21	0.605
BMI (kg/m^2^)	Non-Overweight/obese	1	[Reference]	0.935			
	Overweight/obese	1.06	0.27–4.07				
Smoking	No	1	[Reference]	0.862			
	Yes	0.93	0.42–2.06				
Living with family	No	1	[Reference]	**0.014**	1	[Reference]	0.214
	Yes	0.53	0.317–0.88		0.69	0.39–1.23	
Work	No	1	[Reference]	0.394			
	Yes	1.33	0.69–2.59				
Drink tea	No	1	[Reference]	0.129			
	Yes	3.16	0.72–13.96				
Drink coffee	No	1	[Reference]	**0.020**	1	[Reference]	
	Yes	2.43	1.15–5.14		2.15	0.98 - 4.69	0.055
Carbonated soft drinks	No	1	[Reference]	**0.023**	1	[Reference]	**0.028**
	Yes	3.44	1.187–9.96		3.35	1.14 9.88	
Eaten legumes	No	1	[Reference]	0.403			
	Yes	1.54	0.56–4.20				
Eaten fatty food	No	1	[Reference]		1	[Reference]	0.183
	Yes	1.55	0.94–2.54	0.085	1.43	0.85–2.40	
Eaten fast food	No	1	[Reference]				
	Yes	0.79	0.37–1.67	0.532			
	Out home	1	[Reference]				
Eating style	At home	0.67	0.41 1.09	0.105			
	slow	1	[Reference]				
Eating pattern	quick	1.17	0.72–1.90	0.524			
	Sleep early	1	[Reference]				
Sleep pattern	Sleep late	1.33	0.76–2.33	0.311			

^1^Adjusted for all variables listed in Table 1 or 3; BMI, Body Mass Index; 95% CI, Confidence Interval; OR, Odds Ratio

The bold values signify statistically significant results, typically with a p-value less than 0.05

In the multivariate analysis, subsequent to controlling for factors including age, gender, academic stage, living arrangement, coffee consumption, and eaten fatty food, the consumption of carbonated soft drinks was strongly associated with a higher odd of IBS (OR = 3.35; 95% CI: 1.14–9.88, *p* = 0.028) (Table 2).

### Average marginal effects (AME)

Based on the significant association observed in Table 2, we investigated the AMEs of the potential factors on the predicted probability of IBS adjusted for the confounders in the multivariable model in Table 2.

**Fig. 1:**
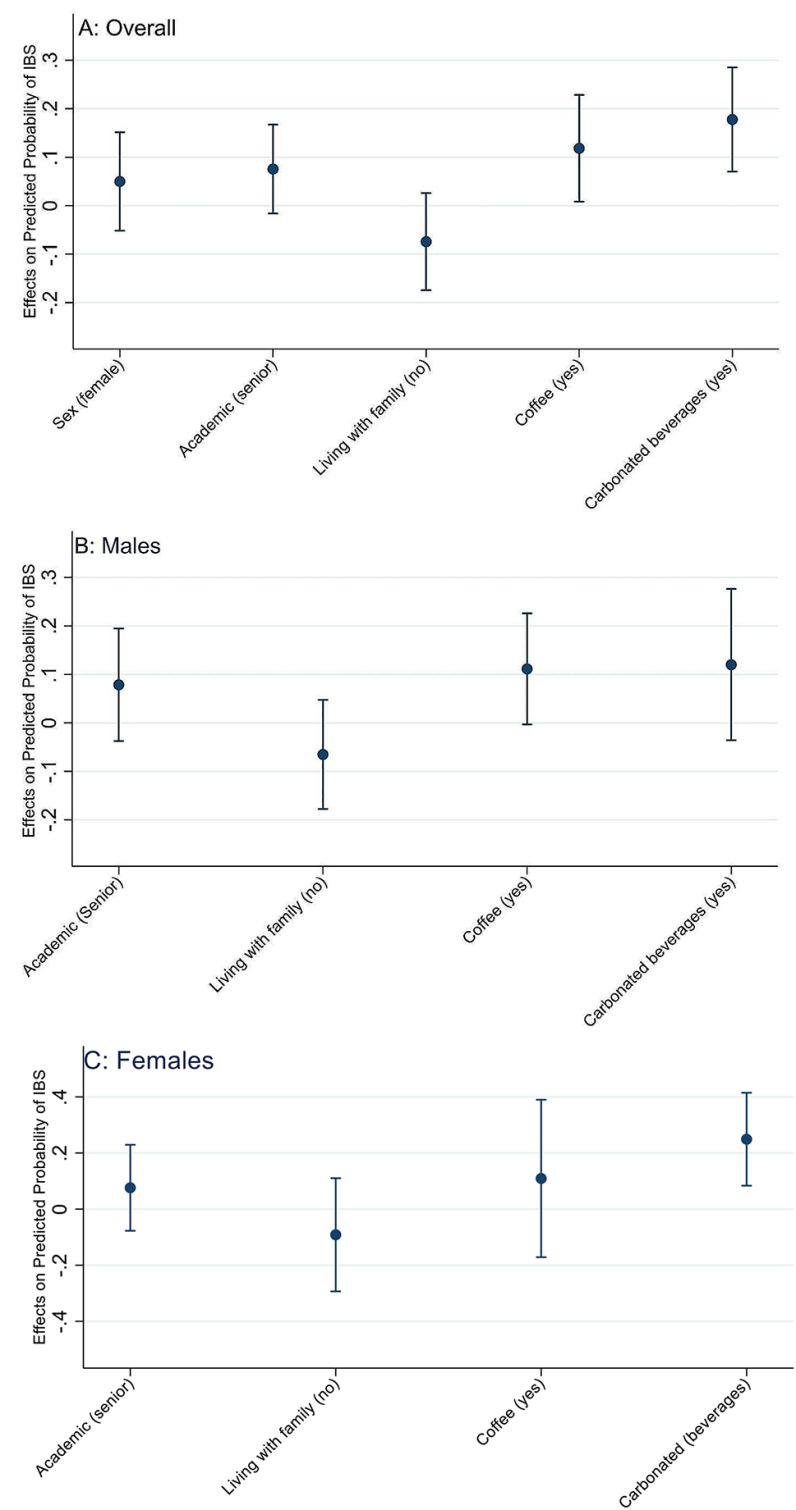
Estimated Average marginal effects with 95% CIs with respect to sex, academic level, accommodation, coffee, and carbonated beverage consumption. **A**: overall; **B**: Males; **C**: Females

Overall, the coffee consumption (AME: 11.84%; 95% CI: 0.83–22.84) and carbonated soft drinks consumption (AME: 17.78%; 95% CI: 7.03–28.53) had a significant effect on the predicted probability of IBS. In males, coffee consumption had a substantial effect on the predicted probability of IBS (AME: 11.41%; 95% CI: 0.32–22.60). In females, the consumption of carbonated soft drinks had a significant effect on the predicted probability of IBS (AME: 24.91%; 95% CI: 8.34–41.48) (Fig. 1).

## Discussion

IBS is a chronic condition involving the gut-brain axis, marked by changes in bowel habits and abdominal pain or discomfort, occurring without any detectable organic disease. To our knowledge, this is the first study to evaluate the prevalence of IBS among Yemeni medical students. The current study used Rome IV to diagnose IBS and identify independently associated factors, providing a platform for understanding the current magnitude of IBS and prioritizing future research efforts.

Our findings showed a high prevalence of IBS among Yemeni medical students, with 26.21% of the participants meeting the IBS’s Rome IV criteria. The mixed bowel habit (IBS-M) was the predominant subtype, affecting 68.48% of the IBS patients. In a cross-sectional study, the prevalence of IBS was 6.6% among medical students, based on Rome IV criteria [18]. Similarly, a study showed that the prevalence of IBS was 7.9% among medical students; based on Rome IV criteria, IBS constipation (IBS-C) was the predominant subtype, affecting 31.5%, followed by the mixed bowel habit (IBS-M), affecting 28.8% of the IBS patients [19]. Another descriptive-analytical study showed that 14% of medical students had IBS; based on Rome IV criteria, IBS constipation (IBS-C) was the predominant subtype, affecting 36.4%, followed by the diarrhoea bowel habit (IBS-D), affecting 25% of the IBS patients [20].

In contrast, the 26.21% prevalence rate of IBS among Yemeni medical students is relatively higher than that found in other regions, particularly Western countries [21]. This elevated prevalence rate may be attributed to the increased stress and burden experienced by Yemeni medical students due to the ongoing war. However, a recent cross-sectional study among Lebanese adults aged 18 years and older reported an IBS prevalence of 46.8%, which is higher than our findings [22]. Discrepancies between these results and ours may be due to various factors: differences in the demographic composition of the samples in the Lebanese study, variations in socio-cultural contexts between the two populations, differences in the study periods, and methodological variances, such as our offline data collection supervised by our research team versus their use of social media for data collection.

Likewise, in another cross-sectional study, 400 medical students were diagnosed with IBS using the Rome III criteria questionnaire. The study revealed that 121 students tested positive, resulting in an IBS frequency of 31.7% among the medical students. However, when examining the subtypes of IBS, it was determined that 26.6% of the cases were of the diarrhoea-predominant subtype (IBS-D) [23].

Similarly, a systemic review study diagnosed 7988 medical students with IBS using the Rome III criteria questionnaire; the prevalence of IBS among medical students ranged from 9.3 to 35.5% [7]. Several research studies alongside our study indicate that the rate of IBS prevalence varies according to the diagnostic tool and countries, with a higher prevalence in diagnosis based on Rome III criteria [24, 25]. In a comparison between the two criteria for IBS diagnosis (Rome III vs. Rome IV), Bai et al. found a substantially lower prevalence rate using Rome IV criteria (6.1% vs. 12.4% using Rome III criteria). They concluded that Rome IV-positive IBS patients represented a subgroup of Rome III-positive patients with more severe symptoms [26]. The relatively high IBS prevalence among medical students may be attributed to their stressful learning environment.

### Dietary factors associated with IBS

Our study found a significant positive association between the consumption of carbonated soft drinks and IBS. This result aligns with previous findings that showed a higher carbonated soft drinks intake among individuals with IBS compared to controls. Additionally, another study reported more gastrointestinal complaints induced by carbonated beverages in subjects with IBS compared to controls. Thus, the higher intake of carbonated beverages may be linked to IBS symptoms due to the presence of caffeine or other components in these drinks.

Furthermore, our study observed that coffee consumption was nearly significantly associated with higher odds of experiencing IBS. This finding is supported by another study that investigated the link between adult coffee consumption and IBS. The study concluded that individuals who consumed coffee weekly or more frequently had a higher risk of experiencing IBS compared to non-consumers [27]. The impact of carbonated beverages and coffee consumption has been linked to the manifestation of IBS [28].

### Covariate factors that affect the predicted probability of developing IBS

Overall, our research established a significant link between coffee consumption and carbonated soft drinks consumption and an increased likelihood of experiencing IBS. Interestingly, this effect varies by sex. We observed that men who regularly consume coffee seem to face an increased likelihood of experiencing symptoms associated with IBS. In contrast, women are more adversely affected by carbonated soft drinks, with these beverages being more closely linked to an increase in IBS symptoms. This disparity might be attributed to ingredients such as sugars or artificial sweeteners found in soft drinks, which are known to affect digestion and nutrient absorption [29]. The findings of our study highlighted the critical importance of individualized dietary guidance in the management of IBS. The findings also suggest that dietary recommendations for managing IBS may need to be tailored distinctly for men and women, considering their differing responses to beverages like coffee and carbonated soft drinks.

### Clinical implication

Our study contributes to expanding the understanding of IBS in medical education setting, emphasizing the importance of considering dietary habits when addressing the prevalence of IBS among medical students. The significant associations with carbonated soft drinks and the near-significant link with coffee consumption suggest that lifestyle modifications could be beneficial. Clinicians can advise medical students to make healthier dietary choices and reduce their intake of these beverages to lower their risk of IBS. This reduction could also decrease the impact of IBS on their education, as symptoms can lead to increased absenteeism and hinder their academic performance.

### Limitations

The study design as a cross-sectional poses limitations regarding the establishment of causality, thus warranting further longitudinal investigations to explore the dynamic nature of IBS within the specific population.

Noteworthy is that data collection relied on self-reported responses, which may be subjected to recall bias and social desirability bias. Additionally, since the study was conducted at a single institution, caution should be considered when generalizing the findings to other populations. Furthermore, it is essential to consider that additional factors not investigated in this study, such as stress levels, exercise, and genetic predispositions, may contribute to the occurrence of IBS among medical students and merit investigations in future research.

For all limitations mentioned previously, we recommend that future research address these gaps and employ longitudinal design to establish casual relationships. Furthermore, involving diverse populations from multiple institutions will provide more reliable data, offering a more thorough comprehension of the complex nature of IBS in medical students.

## Conclusion

Our study revealed a high prevalence of IBS among Yemeni medical students, with dietary factors like carbonated soft drinks and coffee playing a significant role. Notably, these associations varied by sex, with our findings indicating that regular coffee consumption increases the risk of developing IBS symptoms, particularly among males. Conversely, consumption of carbonated soft drinks significantly elevates the risk of IBS symptoms in females. These results underscore the importance of personalized dietary recommendations in managing IBS, considering the varying impact of sex-specific dietary habits. They highlight the need for tailored interventions and heightened awareness of IBS among medical students, emphasizing dietary choices and stress management. Further research is necessary to evaluate how IBS affects the quality of life and academic performance of medical students. Additionally, studies aimed at estimating the prevalence of IBS in the general Yemeni population and identifying other associated factors are warranted to enhance understanding and management of this condition.

### Electronic supplementary material

Below is the link to the electronic supplementary material.

Supplementary Material 1

Supplementary Material 2

## Data Availability

The data that support the findings of this study are available from the corresponding author upon reasonable request.

## Abbreviations

AME: Average marginal effect
BMI: Body mass index
CI: Confidence interval
IBS: Irritable bowel syndrome
IBS-C: IBS with constipation
IBS-C: IBS with constipation
IBS-D: IBS with diarrhea
IBS-M: Mixed IBS
IBS-U: Un-subtyped IBS
OR: Odds ratio
