# Supplementary material for: Dietary habits as associated factors with irritable bowel syndrome among medical students: evidence from a cross-sectional study
Source: BMC Gastroenterol. 2024 Aug 17;24:268. doi: 10.1186/s12876-024-03320-w (PMC11330611; doi:10.1186/s12876-024-03320-w)
Supplement: Supplementary file 1 — Supplementary Material 1 [file 12876_2024_3320_MOESM1_ESM.docx]

**Supplementary File 1: Informed consent**

**The Prevalence of Irritable Bowel Syndrome and Its Associated Factors Among Medical Students at Ibb University, Yemen**

**Greetings, esteemed students,**

Recent studies on irritable bowel syndrome (IBS) have highlighted a significant prevalence among medical students worldwide. Despite this global interest, there remains a notable gap in research concerning IBS among medical students in Yemen.

**Informed consent:**

This study aims to investigate the prevalence and risk factors associated with IBS among Faculty of Medicine students at Ibb University, Ibb governorate, Yemen. Your participation is crucial in shedding light on this condition within our academic community.

The study will collect data by distributing a questionnaire, and participants can fill it out independently. Participants in the study will do the following:

It is filled out with a questionnaire consisting of two parts. The first part will collect socio-demographic and lifestyle information. The second part will include the Rome IV criteria questionnaire for IBS.

The researchers participating in the study also confirm that the study data will remain confidential and will not be used outside the research purposes described in the study protocol.

The study does not expose participants to any risks.

Do you agree to continue studying?

A- Yes ( ).

B- No ( ).

We appreciate your time and consideration. Your contribution is instrumental in advancing our knowledge and potentially improving the health and well-being of medical students in Yemen and beyond.
