# Supplementary material for: Dietary habits as associated factors with irritable bowel syndrome among medical students: evidence from a cross-sectional study
Source: BMC Gastroenterol. 2024 Aug 17;24:268. doi: 10.1186/s12876-024-03320-w (PMC11330611; doi:10.1186/s12876-024-03320-w)
Supplement: Supplementary file 2 — Supplementary Material 2 [file 12876_2024_3320_MOESM2_ESM.docx]

**Supplementary File 2: Self-Report Questionnaire - Part I**

**The Prevalence of Irritable Bowel Syndrome and Its Associated Factors Among Medical Students at Ibb University, Yemen**

**Socio-demographic and lifestyle information:**

Please fill out the following information. Your responses are confidential and will be used for research purposes only.

1-Age: ____________.

2- Academic level: ____________.

3-Gender: ____________.

4 – Weight(kg): ____________. Height(cm): ____________.

5-Smoking habits:

- Do you smoke? Yes ( ) No ( )

- If yes, for how many years? ____________.

- The average number of cigarettes per day: ____________.

6-Accommodation details:

- Type of accommodation: At family home ( ) Away from family home ( )

- Room type: Single ( ) Shared ( )

7 - Employment Status:

- Do you work while studying? Yes ( ) No ( )

8- Eating pattern:

- Primary location for meals: At home ( ) Outside the home ( )

- Average number of meals per day: ____________

- Eating pace: Slow and relaxed ( ) Quick ( )

9-Sleeping pattern:

- Average hours of sleep per night: ____________

- Typical bedtime: Before 22:00 ( ) After 01:00 ( )

10-Fast Food Consumption:

- Do you eat fast food “easily prepared processed food served in snack bars and restaurants as a quick meal or to be taken away”? Yes ( ) No ( )

- If yes, how often? Daily ( ) Times per day: ____________ Occasionally ( )

11-Fatty Foods Consumption:

- Do you eat foods high in fat (e.g., meat, butter, cheese)? Yes ( ) No ( )

- If yes, how often? Daily ( ) Times per day: ____________ Occasionally ( )

12-Legume Consumption:

- Do you eat legumes (e.g., lentils, beans)? Yes ( ) No ( )

- If yes, how often? Daily ( ) Times per day: ____________ Occasionally ( )

13-Carbonated Soft Drinks Consumption:

- Do you drink carbonated soft drinks? Yes ( ) No ( )

- If yes, how often? Daily ( ) Times per day: ____________ Occasionally ( )

14-Tea Consumption:

- Do you drink tea? Yes ( ) No ( )

- If yes, frequency: Daily ( ) Times per day: ____________ Occasionally ( )

15-Coffee Consumption:

- Do you drink coffee? Yes ( ) No ( )

- If yes, frequency: Daily ( ) Times per day: ____________ Occasionally ( )
